# Philosophy in Hospital Architecture

**Published:** 1916-11-04

**Authors:** William A. Pite


### PHILOSOPHY IN HOSPITAL ARCHITECTURE.

By WILLIAM A. PITE, F.R.I.B.A

In the production of the modern hospital, touched by that prevailing spirit happily to be found in most hospitals where self-effacing labour for the benefit of suffering humanity is effected, great gain will be found in collecting individual opinions and objections, which more likely than not are the result of definite personal experience.

This the hospital architect must always recognise, even if many of the sugestions made to him he is obliged to reject. The choice of their professional architect by any hospital committee is a matter of supreme importance, and it should be remembered that competition does not always produce the desired result. That manner of selection has in some cases given an opportunity to a man of hitherto unknown ability; but in many cases it has proved to be unsatisfactory and fallacious. It is also worth bearing in mind that the best architect is not always the specialist, who sometimes lacks personality and a wide outlook when called upon to translate stated requirements into the actuality of bricks and mortar. The best hospital architect will prove to be the man who has over and beyond the necessary acquired institutional knowledge a wide architectural interest and outlook, and one who is not wholly wrapped up in the interesting if unæsthetic details of administrative and constructive economics.

### Artistic Considerations.

Up-to-date hospital construction in the hands of an architect of ability, whose artistic instinct is not dormant, results in refinement without the sacrifice of any fundamental axiom of hospital requirements, and should minister to the enjoyment, mental health, and satisfaction of those whose duty it is to labour therein, or to those whose weary lot it may be to lie for weeks on beds of sickness and pain. Such an atmosphere is essential to every up-to date hospital; and such a sense of repose may be created by the buildings themselves among the inmates, both sick and serving. These characteristics in hospital architecture have been sadly lacking in the past, and attention has been called to the question in the columns of this paper. There is no reason why hospitals should bear the brand of ugliness: no doubt there are difficulties to be overcome, partly due to the regularity of the spacing of windows, openings, and the provision of other features common to hospital planning; but the skilful architect will be able to overcome these difficulties, and, while providing in full for the institutional needs, will be able at the same time to make the elevations consistent with good design. It may be asked how this is to be obtained, and it may seem to be a matter of some difficulty; but modern methods of architectural training have been yielding very promising results in this respect.

Good architecture, for instance, is not necessarily secured by small panes of glass, which some people may think to be indispensable where whole glass is essential for sanitary reasons. We know that plate glass, taking into consideration rounded corners, costs about 100 per cent. more than the best sheet glass; yet this expenditure may be desirable and entirely justifiable æsthetically. From the standpoint also of material it should have an architectural value placed upon it far exceeding that of mere ornament.

### The Use of "Ornament."

Ornamentation should be limited whether outside or inside a modern hospital, and where used should be most sparing in quantity and of the highest quality and refinement. Such repression is most desirable. Modern hospital design should render a definite architectural expression and mark of its own individuality, the outcome of its environment, and as unmistakable to the person of taste as are the ecclesiastical, domestic, or castellated phases of Gothic architecture of whatever period.

Modern hospital architecture must express the nature and purpose of the buildings it represents. This it best achieves by the greatest simplicity of both internal plan and external expression. To attain simplicity and character in any art is one of the most difficult of accomplishments, and is only the fruit of long and close experience, study, and practice. We frequently hear of a successful plan as being "so simple" that any fool could have produced it, so straightforward and obvious. But it is this very simplicity that constitutes the art of architecture. This difficult accomplishment of repression and elimination must not be carried to the point of sheer bareness or ugliness; the accomplished architect knows what is meant by simplicity of treatment, and the lay member of the hospital committee should know and appreciate this quality and demand it.

The architect with the barest materials and methods should be able, in the internal and external treatment of detail of openings without extravagance, to produce by proportion and otherwise an atmosphere which will minister pleasure to all who see it, and by so doing conduce to healthy delight and refreshment. The repetition of ornament externally cannot but merit severe condemnation; and it is a matter for regret that so much shortcoming of this character should have found a place in many hospital buildings, otherwise noble and good throughout the country.

When the architect has been appointed he should be trusted and supported, and should not be subjected to any one dominating influence or personality on the directing committee; he is the servant of all, and he ought to be trusted to secure the best possible results.

In the production of the modern hospital it is essential that the selected architect should be given every information as to the special requirements of the buildings. This will have to be carefully collected, and in most cases enthusiastic physicians and surgeons will be found willing to devote much valuable time and disinterested service to the elucidation and production of data and to prolonged discussions with the architect, which must take place before and during the preparation of the plans and execution of the work. A little company of workers can be elected by the medical and surgical staff who shall form a "watching committee," conferring with the architect and reporting to the staff. These duties are most important, and are perhaps somewhat exacting; but those regularly attending will find much of interest, and will have the satisfaction of knowing that they have definitely contributed to the production of the great work for the benefit of their fellow-men and the advancement of medical and surgical hospital equipment. In some places a professional medical director has been appointed to collaborate with the architect. Whether this is a wholly desirable method of procedure in the production of the modern hospital may be open to question, and should receive due consideration. This procedure generally shifts the main responsibility from the staff who advise the board to the hospital expert. It also relieves the architect of much individual trouble and many anxieties, and in that the appointment has much to commend it. Personally, the writer would value such assistance, but it seems that, notwithstanding its advantages, such an appointment may deprive the scheme of a good deal of individuality, notwithstanding some compensating advantages. Of course, much will depend on the personality both of the architect and of the expert administrator with whom he is associated. Where these two are in sympathetic harmony and in close touch with an enthusiastic staff interesting results may be anticipated.

### Architectural Commissions.

Both in America and Germany it has been the custom for commissions to be appointed, composed of physicians, surgeons, and architects, who have visited certain buildings. It has been the privilege of the writer to serve on such commissions, seeking for and collecting detailed information. Particularly in Germany it may be seen by the character of the detail that the physicians and surgeons have been almost the sole directors, and this would be quite in order provided that it was supervised by an architect who by long experience was cognisant with the requirements. The same holds good to some extent in the United States, and it should be borne in mind that severe climatic considerations rule in the States which create conditions that do not arise either in this country or in the continent of Europe; these special climatic difficulties largely affect the arterial system of heating and sanitation. Except, therefore, in very unusual circumstances where the necessary assistance is not forthcoming from the staff, or in the case of hospitals of special character, it would seem desirable for the architect to seek the guidance of the hospital's staff, who are generally the most distinguished and experienced practitioners of the locality.

An exception might, however, be allowed in the case of the selection of a site: this important question often has to be settled years before any movement in regard to the provision of a building or plans has been made. The architect more often than not finds the site already secured, and it may be most difficult to deal with the important matters of aspect and contour. It is important in a large modern hospital to remember that internal steps on any general level are not permissible. To lay down a hospital scheme to cover a site of from 12 to 15 acres for 600 patients, 300 nurses, and 100 staff, wherein wheeled access to all parts may be secured, is one which will tax the capabilities of the most experienced architect. Hence the great importance of selecting a suitable site which shall yield the best results, such as a fine open south-eastern aspect, with the land, if possible, sloping gently towards the south. The perversity of human affairs more often provides the opposite problem of building uphill towards the south, with various dips, and this suggests contingencies important to provide against when it is possible by forethought in the choice of a site to avoid such a contingency.

### Suggestive Ideas in Existing Hospitals.

Lord Bryce, writing over forty years ago, tells us, "It is indeed true that modern art and literature and philosophy have been produced by the working of new minds upon old materials; that in thought as in nature we see no creation. But with us the old has been overlaid by the new until its origin is forgotten." This is applicable to our great "heap of hospital buildings" of which the London Hospital is a most excellent and striking example. This and other most estimable institutions in London and the provinces, although past the centenarian stage, cannot, by reason of the admirable results that they achieve, be called other than fine hospitals, and are certainly up to date in their treatment of the sick, even if they exist under disabilities connected with out-of-date buildings. But it must be remembered that no hospital remains up to date for long, and that as time passes, even if not relegated to the "heap of buildings" class, they will probably have to stand the "tuning-up" process. They labour under traditional circumstances of a building construction of the past, which included a dislike of light and fresh air.

In such hospitals where there is so much to regret and deplore by the absence of methodical lay-out with cross-ventilation and cut-offs, there is much to learn in equipment, and the earnest seeker after hospital teaching will find these great institutions generally so admirably managed as to be veritable indices of many things he wishes to explore, and full of suggestions. Anyway, the writer has found many excellent arrangements in antiquated buildings, which have proved useful in this way.

Just as new developments in planning are necessitated by the new methods of treatment, it must be through the working of new minds upon the old materials that success will result; and although there may not be a new creation, yet there will be a re-creation which the original designer might fail to recognise or wish to acknowledge. Old methods have been reformed and transformed and overlaid by the new until the original is well-nigh forgotten, and for the better.

### Lessons of the War.

No person interested in hospital planning should neglect to visit any hospital he comes across, be it good or bad. Intercourse with the working staff will reveal points of failure and defects which can be either avoided or remedied in actual practice. It has been stated that architects have gone as far as it is possible to go with the development of hospital planning. This is by no means an accurate estimation of the position; there is much yet to be done in the way of simplification of detail, and it is only by the close and constant study of these matters that definite advance can be made. The future possesses much for us; sooner or later the experience at the numberless base and temporary hospitals will be available. Here, under temporary conditions, experimental arrangements which are not desirable in general hospitals can be tried and expedients adopted to meet immediate needs impossible in normal times, and experiments in sanitary methods and disposal have been tried and adopted. The great base camps at the Front are marvels of organisation, and should be reported on by Army experts, so that the lessons of the great war may benefit mankind and advance hospital development. We have to learn what our Allies have done, and, further, what our enemies have accomplished, and they are no mean organisers in such matters. The experience of the Franco-German War had a wide and far-reaching effect upon German hospital architecture, which is a State organisation, and indirectly on our own. The co-ordination of these and kindred matters will call for the most assiduous scrutiny if the British hospital is to hold the supremacy which we believe it holds to-day.

While strict economy is essential in all hospital building, and will be necessary in the future, it must not be thought that cheap construction is possible. The production of bare surfaces, the avoidance of right angles, ledges, hollow places, and projections of all sorts, are fundamental axioms of good construction; but it must not be thought that they are inexpensive, for they are not. Often this doctrine is overdone, yet it is essential to cleanliness and to the well-being of the patients. It is of the utmost importance to consider economy of labour in hospital administration, and here money may be well expended on anything which tends to the reduction of working expenses and of the cost of upkeep of the buildings.

### The Modern Ward.

In the evolution of the modern hospital the chief units which exhibit most variation from older types are the wards. From back-to-back walls with a mid-wall between, or four wards traversed by a corridor lighted only on one side without any attempt at cross-ventilation, with small and inefficient sanitary annexes attached thereto and a number of other disabilities, the advance to side-lighted, cross-ventilated wards in recent years has been rapid. In the Diamond Jubilee year a special Hospital Sunday Supplement of this Journal appeared, in which there was an interesting review of hospital progress in Queen Victoria's reign. This included an inset of photographs entitled "Then and Now," illustrating one of the wards of St. Thomas's Hospital before its removal to Westminster, and a ward of 1897 from the Homœopathic Hospital in Great Ormond Street. It was there noted that "comparison shows very strikingly the sentiment, if we may so call it, which has gradually converted the severe barrack-like apartment into a delightful haven of rest for sufferers, almost palatial in general appearance yet homely in the comfort it affords."

### Suggested Design.

Over twenty years have passed since the second of these hospitals was designed and erected, and much progress has been effected since then, although the arrangement has remained generally unchanged. The modern ward of, say, twenty-four beds, rectangular in plan, should be designed approximately as follows. Adjoining the main corridor, approached therefrom possibly by an archway, it may have on either side a day or convalescent room, which on occasion may serve the purpose of a class-room, while on the other may be the sister's office, with w.c. and lavatory for the nursing staff attached. Across the ward corridor at this point will be the first pair of swing doors isolating the cut-off from the main building. This part will be about 7 feet 6 inches to 8 feet high, thus affording a clear blow-through above and below of about 4 feet in height. This central cut-off space should have adjustable windows, preferably of metal, on either side. This cut-off space might be octagonal in plan but possibly unequal in the length of its sides, the doorways being arranged on the four splays opening therefrom, which is a very convenient arrangement for getting trollies in and out.

A similar arrangement is necessary for single-bed wards, the doors of which should not be less than 3 feet 3 inches in the clear and the corridor not less than 8 feet wide, otherwise there will be difficulty in wheeling a patient's bed in and out. Where a corridor is less than 8 feet wide the door to any room receiving a bed must be on the splay. Swing doors, as has been noted, complete the air lock. From the cut-off space entry is gained to the sink-room which serves the single-bed wards, and also the scrubbers' room, without entering the sick-ward corridor proper. Having entered this, on the east side may be the patients' clothes store, which most hospitals find it essential to have on the ward floor after disinfection has taken place. This apartment should be warmed and have ample light and external ventilation. The duty-room, with a food larder adjoining having external ventilation, should have fittings carefully designed for the purpose, amongst which may be a fruit cupboard, with compartments for each patient. For cleaning purposes it is convenient to have all fittings such as dressers mounted on Slingsby's castors, so that they may be easily moved any way. A spacious clinical-room is essential to the up-to-date hospital, and this should be reached both from the corridor and from the ward. Here will be kept the various lotions and the poison cupboard.

The opposite side of the corridor will be occupied by the single-bed wards and the linen-room, the fittings of which will require careful arrangement. The doors should be flush and solid, not less than 3 feet 3 inches wide in the clear opening, and with neither brass nor gun-metal fittings to clean. Over the doors and elsewhere should be glazed fanlights in metal frames. The universal adoption of fanlights over all doors may with confidence be recommended; for it is a most valuable adjunct in the cross and general circulation of air, which is kept in constant motion in the corridors automatically by the swing doors and also by longitudinal ventilation across the blow-through. The double swing doors can conveniently be of long glass panels, and have a very pleasing appearance with white metal cross-rail and kicking-plates. The old system of sunk spring hinges in the floor must not be considered. The springs can quite easily be arranged in the sills of the doors, and can be as readily reached; when open to the full extent they should so remain without the assistance of hooks or fastenings. Over the fanlight to the ward door should be a double-faced clock worked from a standard dial in the central station. The doors to the wards might have clear glass peepholes. The ward proper should have no projecting piers, and the window faces should be practically flush with the walls; there will therefore be no internal sills. The ceiling, likewise, should have no projecting beams and be level throughout.

### Sanitary Towers.

The principal heating should be by central fresh-air stoves, an essential factor for ventilation when windows cannot be opened. In extreme weather this heating can be assisted by radiators fed by fresh air. Two positions alone suggest themselves for the sanitary towers: these may be at the far end, splayed in plan, amply cross-ventilated, and with a balcony between forming a protecting sun-trap. This method has been highly developed and is now yielding to various improvements. Sanitary plumbing has made such progress that it is not now considered necessary to cut off such apartments as bathrooms, which are more conveniently placed at the home end of the ward, with a sanitary tower adjoining thereto; but this must not materially interfere with the early morning sunshine. This tower will demand very careful planning. There is much more which could be written about wards and their fittings which must be left for a future occasion, but enough has been said to show how great the advance is when wards can be heated without running the flow and return pipes through them, and in securing adjustable louvered sash windows which obviate the use of the objectionable hollow boxings.

### Nursing and Household Staff.

Many changes are taking place in the housing arrangements of the staff who are in daily residence for practically the whole year, with the exception of a brief annual vacation and an occasional day and night off. Each sister and nurse must have her own bedroom. How different from the curtained or boarded cubicle which has done and, alas, in some cases still does duty for " an own bedroom "! The rooms may have a wardrobe built as part of the structure, with drawers and cupboards, and bevelled plate mirrors in the panels of the wardrobe doors are highly appreciated. The heating and ventilation is best arranged to meet the individual needs by collecting the cubic capacity of heating required in the corridor, where abundant fresh-air ventilation should be provided by bays, so that the corridors perform the duty of ducts. A fanlight is provided to each room, and the occupant can control the quantity of warmth, while the extraction is provided by a flue at the floor level, which runs to a chamber in the roof cleared by extraction fans. A certain proportion of rooms may have fireplaces, and a sick nurses' floor as a separate department is in every way desirable, where the patients can be entirely separated from the staff for quiet, and apart from the night nurses. There are many other considerations relative to nurses' homes which it is impossible to enlarge on here.

Large institutions are little self-contained worlds of their own, and they have a peculiar attraction for some domestic servants. It is necessary that a hospital should have thoroughly good servants. This can only be secured by catering for their comfort. The bedroom is the key to the position. Give the maids the privacy of a self-contained apartment and continuity of service will no doubt result. This can be done and cross-ventilation secured by leaving the upper part of each room open for a space at the top; ample provision of baths should complete the section. The porters and orderlies will be dealt with in the same way, but in another part of the building, and the particular comforts of the men should be anticipated. In all these departments much advance has been made, and will still further ensue in the days to come.

### The Commissariat Department.

It would take some pages of this journal to describe and illustrate adequately the requirements of the commissariat department of a large modern hospital. The modern hospital kitchen has come to its own, but it can only retain that position by the loyalty and devotion to duty of the staff. It should not only be spotless, but it should also be the showplace of the hospital. Its sculleries, larders,

(*Continued on p.* 95.)

### PHILOSOPHY IN HOSPITAL ARCHITECTURE (*continued from* p. 92.)

fish, vegetable, and other stores, pastry kitchen and flour store, arranged to be under the control of the cook in her office, are grouped around the kitchen.

This department is usually covered with a steel-trussed roof, with innumerable ledges for the dust, dirt, and grease which gather in an amazing way upon its walls and ledges when vaporised. The top-lighted roof should be open plastered and painted, without bars or rods; the ventilation ducts for extraction should be partly mechanical. The storage of the food and hardware are matters of much interest and importance, and most large hospitals have very capable sister-housekeepers, whose delight it is to keep the department at a high state of efficiency. The fittings, the cupboards, the hinged bins for storing rice, tea, coffee, and other groceries are many and detailed.

Co-ordination in planning is very necessary in this department in order to secure quick and efficient service to the various departments, so that as little delay as possible may result during the passage of the trollies to the several lifts. It is found possible to arrange for special heating by means of electricity in connection with the trollies. This has already been effected at the entrance to some wards in London; but it is not absolutely essential where efficient hot-water jackets are provided.

Fittings and Equipment.

The vegetable stores should receive due consideration, so that the bins with drop fronts can be kept fresh and clean and the stores themselves tidy. Nothing contributes so much to efficiency as to have fittings in all departments which are the result of practical experience. The aspect and position of the various larders, with a proper cold room, the latter cooled from the refrigerating plant, should be in due relation to the kitchen, around which the several departments are arranged. In connection with this there should be a suitable fish store with glazed water-tanks and glazed slabs for preparation. The sculleries should be provided with labour-saving appliances planned for actual service, due regard being paid to lighting, ventilation, and working space. Such details as, for instance, dealing with earth-laden water from potato-washing sinks may call for attention. The pot scullery is a separate department, which will best adjoin the large kitchen, and should be, as all other departments, pleasing to the eye. A pastry kitchen is also necessary, distinct from the main kitchen, for the preparation of pastries, sweets, etc., and must have its own flour store, larder, etc. This section should have a separate approach other than through the kitchen. Near the cook's office, which should be placed in a central position in the department, should be the grocery and other stores which may be needed for the departmental service. The position of the milk store is of importance; cooling, Pasteurisation, storage, and dispatch can only be mentioned to indicate the extreme importance of the department.

A central position should be provided for the steward's office, the opposite sides of which should be glazed, which will be found to be desirable for supervision. Such an arrangement as this permits the daily food and other stores to be received and checked on one side; while the passages should be of ample width. A similar arrangement can be made on the opposite side for the reception of the hardware and other goods, which, after checking, are passed through to the general stores. The unpacking yard should be partly covered for service.

Provision is necessary for the casual services of the hospital in the employment of scrubbers and others. For these indispensable workers a fair-sized room should be provided, with lavatory accommodation. Each worker should be provided with her own cupboard for hat and coat, pail, and other implements of service, planned to be accessible, visible, and readily cleansed. There should be a separate room for mess. The access to and egress from this little section should be direct, and pass the steward's office, by which means prevention or detection of possible pilfering may be under observation. This point is emphasised here to indicate an underlying principle applicable to every department concerned.

Almoners' Department.

A little over ten years ago the almoners' department made its appearance in hospital work. At first a single room was considered sufficient for the new organisation, which was under the direction of a lady almoner. This department has fully justified its existence. The utility of the work very soon outgrew the single room, which has now become, certainly in hospitals of magnitude, an indispensable department of service, with its own suite of rooms or offices. The best position for this is a central one, and must be on or be adjacent to the route to the out-patient dispensary waiting-hall. All new patients on their first visit must interview the almoner before they obtain the medicine or medicament prescribed in their case-book. The department may be planned off a wide corridor with benches along the wall, or with a small hall. The former arrangement is perhaps preferable. Half a dozen or more small offices may be necessary; intercommunication for consultation being provided. The most important room, to which all the almoners should have ready access, is the card-index room; a tea-rest room and lavatories should be provided, and, if practicable, a separate exit. The whole should be in telephonic communication with the out-patient department.

Conclusion.

The foregoing pages have briefly and inadequately set forth what, in the writer's opinion, underlies sound and efficient hospital architecture. In this it has been noted that the plan and detail of arrangements should not be dissociated from artistic considerations, though the actual practical needs must be the *raison d'être* of the whole scheme. The most important qualification for the hospital architect is patient and tireless research, which will call for years of study of the essential qualities of simplicity and directness of treatment, which alone can produce qualities of real distinction. Enough, it is hoped, has been said to stimulate interested study on the part of architects who have had some experience of hospital planning, and to afford suggestive lines of thought to administrators and members of committees who may have to undertake the responsibilty not only of selecting an architect, but also of directing his operations. There is hardly an old existing hospital where something helpful in arrangement in detail cannot be found, and there is certainly no hospital which can go for long without it being found necessary to execute some manner of improvement dictated by the progress of science or to provide for some obvious improvement or a new treatment. In such a contingency the hospital direction will be well advised to secure the best experienced architectural advice obtainable.

